# Diagnosis

**Published:** 1878-09

**Authors:** L. C. Ingersoll


### THE DENTAL REGISTER.

VOL. XXXII.] SEPTEMBER, 1878. No 9.

### DIAGNOSIS.

BY L. C. INGERSOLL.

*Read before the Iowa Dental Society.*

Diagnosis is the art of detecting disease through the interpretation of symptoms. Anything is a symptom that foreshadows, is the result of, or in any way is a manifestation, expression or sign of disease.

Diagnosis is the most comprehensive of all science relating to the practice of dentistry. It includes pathology because it is a careful application of the principles of pathology to specific cases. It includes anatomy, because disease is localized in organs, structures and tissues of the body—effects contiguous parts and traverses to remote parts over nerve and vascular connections, which must be understood in detecting disease. It includes physiology, for no one can detect deviations from normal conditions and functions unless he knows what normal function is and knows well the economy of life and health. It includes all except therapeutics, which is the next step beyond, and is one of the dependencies of diagnosis. There can be no rational treatment or cure of disease without a correct diagnosis.

The word diagnosis is derived from two Greek words—*dia*, through, and *ginosko*, to know—to know through. It implies a knowledge that penetrates—a perception of the inside as well as the outside of things. Practically considered, the term means, *know your case, know it thoroughly, know it through and through* before you attempt to treat it. No word could have been chosen to denominate this branch of science which could be more clear, pointed or technical, or have a more imperative force than *diagnosis*.

To gain a clear and comprehensive meaning of the word is to gain a high conception of the importance of the science, and a prompt suggestion of the method of far too many failures.

The most common and plainly discernible symptom of disease is pain. Yet the cases are numerous of disease not attended with pain. I might employ a tautological expression and say that disease is the most common symptom of disease—using the word disease in its two senses, its technical and its elementary sense. The technical word disease is the compound of two words, dis and ease, which mean want of ease or discomfort. To say, therefore, that dis-ease or discomfort is the most common expression of disease would be both true and forcible. Want of ease or discomfort is the negative expression of pain. Increase the discomfort, and pain in a positive form is sensibly manifest.

Pain is so common an attendant of disease, that in its absence other manifestations are quite often overlooked, and its presence is often misinterpreted.

This leads me to suggest a caution not to trust sensation too implicitly as a guide to diagnosis. Take a simple case of dentalgia. A patient comes in complaining of tooth ache in one of the inferior bicuspids. He is certain concerning the location of the pain. On examination you find the tooth but slightly decayed and question the possibility of pain arising from it. But should you trust to the sensation experienced, and without further examination dismiss your patient as having only a case needing the operation of filling, you might in so doing make a serious mistake. Had you made further examination you probably would have discovered exposure of the pulp of the second or third molar as the source of the pain and find an explanation of the pain in connection with the bicuspid, in the fact that the dental nerve supplying the molars sends a branch through the mental foramen located just below the root of the second bicuspid, and is distributed outwardly to the lower lip. By inflamatory action in the mental artery the nerve has become constricted at the point where it passes through the outer plate of the maxillary bone and causes this bicuspid pain.

Again, take a case of determining the vitality of the pulp by refrigerants—a drop of water or a piece of ice is placed upon a tooth. The excitement of pain under such a test is not alone sufficient evidence of vitality in the pulp. Should your patient be of a decided neuralgic diathesis, cold water could scarcely be tolerated on any of the teeth; even pulpless teeth are sometimes so susceptible to its influence as to deceive both the patient and the operator concerning the character of the sensation experienced.

Again, nervous fear deceives with the apprehension of pain and is attended with all the outward signs and demonstrations of pain, when no physical pain is actually experienced.

Suppose you attempt the test of vitality by drilling into the dentine. In many cases the trembling fear that the instrument *may* suddenly plunge into the pulp chamber produces in the mind of the patient an impression akin to that of actual pain, and pain is reported though it may afterwards be found to have been but the exhibition of fear.

Drilling to test the vitality of a tooth may deceive also in another way—by the manifestation of pain even though the pulp be entirely wasted.

I know, we are in the habit of attributing all sensation in the teeth to the presence of the pulp. But histology reveals other vital connections with the hard tissue so intimate in their relations to the pulp that the *dead line* of pulp extintion is scarcely to be traced in the dentine.

It is not uncommon to find the dentine tubuli opening into the canaliculi and lacunæ of the cementum ; thus forming a vital connection between the cementum and the nerve plexus that is spread like net work over the periphery of the dentine. If then the pulp has been devitalized without exciting any serious peridental disturbance vitality may yet remain in the uncalcified portion of the nerve plexus that underlies the enamel, after the pulp chamber and the root canals have been entirely vacated.

Sensation on the entrance of the drill is not therefore a certain evidence of the presence of a living pulp.

This vital connection of the dentine with the cementum, is an important fact to be remembered in estimating the vital support and longevity of a tooth after its pulp has become extinct.

I desire to call attention to the very critical judgment required in a case like the following: A cavity is prepared in the anterior portion of the mouth, and no suspicion has been aroused of approach to the pulp. The rubber dam has been adjusted and a thorough cleansing of the cavity accomplished, the foil is prepared, and as your patient sees the gold about to enter the tooth he enters his mild protest by the remark "Doctor it aches."

For a moment the gold cleaves to your tweezers while you muster your ætiology for all possible causes of dentalgia, and one by one they are applied to explain the manifestation of pain. Should you conclude that, although you had not before discovered it, the pulp must be nearly or quite exposed and need capping, your operation might be entirely successful while your diagnosis is entirely wrong. And as for the operation of capping it would receive unwarrantable credit.

Should you conclude it to be wisest to delay the operation a few days for topical treatment and apply anodynes, escharotics, astringents, etc., the pulp might survive it, and the after operation of filling be painless and every way successful, yet your diagnosis be very wrong.

Should you conclude that to make the operation safe, the pulp must be extirpated before filling, your whole operation might be successful as a dernier resort for the preservation of the tooth, and still your diagnosis be wholly wrong by mistaking the cause of the sensation of pain.

To arrive at a correct diagnosis of the case, consider the changed relations to its nominal surroundings. The natural protection of the lips has been removed. The warm influence of the saliva has been excluded by the rubber dam. That protection afforded to the dentine fibrils by the decalcified dentine has been removed by the work of excavating, and nothing prevents the air of the room—possibly the air from an open window—from direct contact with the cavity. Under these abnormal conditions and by this means the temparature of the pulp is suddenly reduced several degrees below its normal temperature, and the sudden vascular contraction thus induced causes the pain.

It is evident therfore that this sensation of pain is no evidence of disease in the pulp, and therefore needs no therapeutical treatment. And there being no exposure of the pulp and no near approach to it, the case needs no capping. Much less are there any conditions present that indicate extirpation as a remedy. A rapid filling and restoration of the the tooth to its normal environment, restores the normal temperature, relieves the pain, and all is well.

I have said that diagnosis must precede all rational treatment. It must also accompany treatment to the end—until health and soundness are recovered. Under treatment there is a series of modifications of conditions indicated by changed symptoms. These modifications must be observed with the same critical care as were the first developments of the case. In this connection I desire to call attention to the deceptive influence of anodynes, narcotics, and antiseptics.

To obtund nervous sensibility is an immediate relief of pain. So also is a removal of the exciting cause a temporary relief. But neither is a certain evidence of remedial effect on the disease.

Many a man has taken copious drafts of whisky to cure the toothache. He may feel the pain less though the disease steadily goes on. With carbolic acid and creosote one may continue to obtund the sensibility of a tooth till the pulp, being but half alive, will be obtunded to death. The treatment having the effect to cause a painless death of the pulp, may be interpreted to have restored the pulp to a condition of health—and therefore painless, Of course to fill a tooth under this false diagnosis would be disastrous.

Suppose a patient comes into the office complaining of slight pain in a molar tooth. There is a small opening in the central depression in the crown, and yet spreading under the enamel, yet not involving any considerable portion of the tooth substance—has never ached till within a day or two, and at no time has the pain been severe—is worse after eating than at any other time—after picking the food from the cavity the pain ceases.

You hastily remove the contents from the cavity—syringe with warm water, apply a pledget of cotton moistened with carbolic acid and dismiss your patient to call again in one or two days. On his return he reports having experienced no pain since the former treatment. You consider the report favorable and continue the treatment. Sealing up the cavity you dismiss him for two or three days. After this, the pain not having returned, you dismiss your patient for a longer period—not wishing to over medicate—but advise an immediate return to your office on a recurrence of the pain. There is no recurrence of the pain, and at the time appointed for the next visit you determine to fill permanently. You are delighted to find the bottom and the lateral walls of the cavity firm and hard and free from painful sensations on touch of the instrument. There being no peridental inflammation, neither pressure nor malleting, causes any flinching. The operation is completed and the patient goes his way—but to return again complaining of pain worse than he has ever experienced before.

What is the matter? A false diagnosis from beginning, and self-deception by the antiseptic influence of carbolic acid.

A true diagnosis of the case might be thus stated: The tubuli of the dentine intervening between the cavity of decay and the pulp cavity were vacant by complete solution of the fibrils that have occupied them. The pulp itself was in a state of suppuration and slow decomposition. In the decomposition gases were formed filling the vacant portion of the chamber and being emitted through the open tubuli. When these gases could pass out as rapidly as formed no pain was felt. When the exit was impeded by foreign substances accumulated in the cavity of decay, the expansive force of the confined gas created pressure upon the vital portion of the pulp and caused pain. The more intense pain after eating was occasioned by the more complete stopping up of the cavity by the compressed food. When the food was picked out the confined gas by force of expansion, passed slowly out through the open tubuli and relieved the pain by relieving from pressure the living portion of the pulp.

When therapeutical treatment was resorted to, the cleansing of the cavity gave free egress to confined gas and whatever solid matter was put in solution by decomposition, and the antiseptic influence of the carbolic acid arrested so completely the decomposition of the pulp that whatever gas was formed, readily made its escape without producing any reactionary force upon the pulp, and thus the pain was entirely arrested. Mark, I say the *pain*, not the disease, was arrested. The continued treatment simply retarded the disintegration of the pulp and rendered the process painless.

When the operation of permanently filling, was performed antiseptics could no longer be introduced, the decomposition was renewed with vigor, and there being no possible escape for the resulting fluids and gases the pain necessarily became intense.

There are probably no greater failures in diagnosis than are made with regard to the pathological condition of the dental pulp. This delicate organ is so concealed from view and its manifestations of disease so peculiar and obscure that a correct observation of symptoms is extremely difficult, and great allowance must be made for errors of judgment concerning its pathological state. Experience is a large factor in correcting such errors.

When the pulp of a tooth has become exposed to external influences a series of pathological conditions in more or less regular succession may be considered inevitable, unless the disease is arrested. Our knowledge of the pathology of the pulp must depend upon our knowledge of the anatomy of the organ, and of general pathology in relation to similar structures. The function of the pulp is so limited and so unimportant relatively to other vital organs that functional disturbance under disease can seldom be made a matter of observation. While on the other hand the limited vitality of the pulp, its slight tenacity of life, its tendency to obliteration, all point of structural change of the organ under disease; and this is no doubt the tendency in all cases of exposure.

Before proceeding farther with this branch of my subject, I cannot forbear to express my great surprise that the profession have so generally ignored the necessity of diagnosis as a preliminary step to treatment of an exposed pulp. In the highest representative bodies of the profession as well as in those of lower standing and authority, you may listen to discussions lengthy and learned, concerning treatment of exposed pulp, and hear almost nothing said of the varying conditions of the pulp induced by the exposure, and needing treatment according to the varying pathological conditions.

You may listen to long essays on "Exposed Pulp," describing treatment, without hearing scarcely a word about any treatment peculiar to this or that condition of the pulp. You may read lengthy articles in the journals, all about exposure, without even naming any other abnormal condition than exposure, or even proposing to treat any other condition than exposure. I am glad to notice an exception to this in the last *Dental Cosmos* (July) in an article by Dr. A. H. Thompson, of Topeka, Kansas.

How are we to interpret such silence? Do the profession esteem diagnosis of the pulp a matter of any consequence? Is the same treatment adapted to all conditions? Do the words "exposed pulp" have a meaning so comprehensive as to cover all pathological changes through which the pulp may pass from normality to complete dissolution? The glossary of pathology contains no such form of words as "exposed pulp." The term "exposure" implies no form of disease and recognizes no pathological conditions of the pulp.

The word conveys no other definite idea than a mere mechanical opening and does not necessitate or force upon the mind any idea of pathology. Yet this word has come into general use, not to express pathological conditions, but to cover up the worst forms of disease. The word itself caps the climax of all deceit concerning the pulp. It is a mind-capper, a thought-capper, a knowledge-capper. Is it any wonder then, that led by the mere mechanical idea which the word expresses, the profession should so generally have adopted the practice of a simple mechanical capping for a simple mechanical opening? Is it any wonder that a mechanical capping should come to be considered the *summum bonum* of pulp therapeutics?

Mechanics could play a brilliant part with anatomy if pathology could be put under bonds to keep the peace. It could not only cap the opening into the pulp chambers, but could splice the pulp and lengthen out a nerve with gutta percha. But pathology will not keep the peace under such circumstances.

How to treat an exposed pulp, has been *the* problem before the dental profession for the last twenty-five years. We have made some progress towards its solution, even great progress. But, as it appears to me, one capital mistake has been made, and that is, a failure to diagnose the case of exposure with regard to the pathological conditions of the pulp which invariably follow exposure.

An exposed pulp may be found in any one of the following conditions, each of which defines a functional or structural change, and indicates a treatment successively modified to adapt the remedy to the changed condition of the diseased pulp.

However the exposure may be produced, whether suddenly, by the excavator, or by the slow process of decay, the first effect is an *irritation of the pulp.* Its normal condition is that of confinement and exclusion from the air and from the fluids of the mouth. By exposure the atmosphere and saliva, both foreign elements are brought in immediate contact with the pulp. The nerve filaments of this delicate organ are first to respond in twinges and flashes of pain as the direct result of the irritation.

The irritation continuing, a second condition is induced which we call *inflammation.* This is a response from the vascular system of the pulp. Irritation and nerve excitement have accelerated the flow of blood. This early observed fact was, by the old latin authors incorporated into a maxim thus: *ubi irratio ibi fluxus*—where the irritation is thither flows the blood.

Inflammation, therefore, is characterized by quickened arterial action, increased quantity of blood flowing into the pulp, and consequent distention of the vessels, and heat.

But the susceptibility of the blood vessels to distention, is limited. The contractil power of the arteries is diminished by being overtaxed. The capillary vessels fail at length to pass the whole amount of blood over into the veinous system. Hence the blood accumulates in the pulp in greater quantity than is demanded for normal nutrition.

This condition of the pulp is known as *congestion*, and is the third to be named in the progress of the disease.

Trace it still farther and we find that the blood thus accumulated has wasted its vitalizing influence and become degenerated, and must be disposed of. Not being able to pass through the capillaries it bursts their weakened walls and passes to the the pulp as pus. We now have a fourth condition—a *suppurating pulp.*

If perchance this does not happen, and the capillaries are not distended and disposed to pass the accumulated blood, this superfluous amount of blood may form new and superfluous tissue, thus expanding the pulp and forcing it out into the very opening caused by the decay. This excessive nourishment develops a condition known as *hypertrophy* of the pulp. It is the direct opposite of and antogonistic to the condition last named, and the fifth condition possible. Pursuing this line of progress we find that hypertrophy, which is an excessive development of normal tissue, tends to the development of new tissue of another kind—a *fungus growth*—called sometimes *polypus* or *tumefied pulp*. This is the *sixth* condition in order, although this tendency of the pulp is not very common. I have met with several cases in my practice, in which the abnormalities had adapted the pulp to an open air life free from pain—the excrescence filling the whole cavity of decay, and overhanging the margins. This tumefied condition is highly vascular and bleeds freely on the slightest touch.

Returning now to the suppurating pulp we find it undergoing a destructive process both of the functional life and of the substance of the pulp. Its cell structure is being broken down. The peripheral cells, which are the working cells, *odontoblasts* as they are called, being destroyed the closing of the exposure by a deposit of secondary dentine if not impossible in the nature of the case is altogether improbable, and the wasting away of the substance of the pulp reduces it to a fraction of its normal size and a fraction of its normal vitality. It is alive, with a tendency to death and obliteration. This is an emasculated pulp, a fragmentary pulp, and is the seventh and last of the pathological conditions I have to name.

Now let me ask the thoughtful practitioner, if, in view of the definite and varying pathological conditions which I have named and to which an exposed pulp is subject, is it enough for the purpose of rational treatment, to know simply the fact that the pulp is exposed? Again let me ask, is it in the nature and the therapeutical power of carbolic acid and oxychloride of zinc to cure each one of the seven forms of pulp disease which I have named and restore the pulp to its normal functions? I believe the negative answer is the only intelligent reply that can be given to these questions

If so, every case of pulp exposure demands most imperatively a critical diagnosis of pulp disease.

When then I am asked concerning the treatment of exposed pulp, the question is not an intelligent one, and can not receive an intelligent answer without a thorough diagnosis of the diseased condition of the pulp which shall determine whether it is in a state of irritation, inflammation, congestion, suppuration, hypertrophy, tumefaction or emasculation.

Admitting that such a diagnosis is extremely difficult, without it all treatment is a random effort, and mere guess work. I had intended giving some of the more marked characteristics by which these several conditions may be known. Thus to stimulate and encourage study and observation of the nature of pulp disease. But to avoid wearying you with too great length of my paper, I feel compelled to confine myself to general principles and omit, for the most part, minutiæ.

There is one department of my subject to which I have not yet alluded, and that is "prognosis," which is a judgment of the course of disease with regard to its probable termination. It seems to have been assumed by many that orthodox treatment of any case of exposed pulp is certain to restore it to health.

Within a few years some have been inclined to doubt the succees of their own and of others treatment of many bad cases which they have been accustomed to treat, and have narrowed down the list of cases of permanent success to that few which exhibit the most favorable symptoms. But the capping process, and the assumed preservation and the life of the pulp have been so commonly made the criterion of judging a man's professional skill and standing, that very few have dared to express their doubts or reveal the modification of their practice.

Doubting has sprung up from the multiplied and increasing number of failures. But, naturally enough, may have accounted for the failures not from anything in the nature of the cases but from some presumed faulty method of applying the treatment. But the more we study the nature of the pulp, the peculiarity of its functions, and its relations to surrounding tissues, the more plainly it will appear that failures do arise from the very nature of the case.

Diagnosis of every case of pulp disease with reference to treatment should be made in full recognition of the following facts:

First. The pulp in its most healthful physiological state tends to obliteration.

Second. The vitality of the pulp is so feebly maintained in mature teeth that the slightest causes often produce death.

Third. When diseased, the natural tendency of the pulp to extinction is greatly enhanced.

The first fact just stated is worthy of the most thoughtful consideration in relation to prognosis. In a paper which, by request, I presented before the Odontological Society of New York city, at their meeting in April last, on the *Relations of the Dental Pulp to the other Tooth Tissues*, I adduced a large number of illustrative facts drawn from animal physiology and instituted a line of argument based upon well observed facts in human anatomy and physiology as applied to the genesis and exodus of the teeth, in proof that the pulp even during the period of its most legitimate performance of function *tends to obliteration.* Without again offering the proofs I must refer you to the proceedings of the Odontological Society, published in the *Dental aud Oral Science Magazine*, where I have at some length discussed this and other important relations of the dental pulp.

I shall therefore in the present paper assume it as a fact, and call your attention to it as all important in diagnosis of pulp disease.

The question now plainly resolves itself into this: with the natural tendency of the pulp to obliteration how great reliance can be placed on its waning vitality under disease to restore the organ to healthful and permanent performance of function?

This natural tendency exaggerated by disease must be overcome as well as the disease itself. A rational diagnosis of disease of the pulp must therefore include an estimate of the vital force yet remaining in the pulp after having greatly exhausted its function in the formative work of organizing the hard tissue and having exhausted its vitality in combating disease.

A physician does not deem it necessary in every case to consider the disease with reference to prognosis because he expects to control the prognosis by his remedies. But when the disease and the action of remedies is modified by constitutional idiosyncrasies, peculiar diatheses of heredity, it is all important that prognosis should enter largely into his examination of the case. In diagnosis of disease of the pulp one all important and ever present fact must be considered and must have its influence in controling the judgment of every case. And that fact is not an idiosyncrasy, or any peculiarity of one case as differing from another in any manner, but it is a fact having all the force and power of a law of nature. It is a law of nature. The gradual extinction of the pulp as life advances is not the result of the disease, but it occurs as a natural process of tooth development; it is the uniform working out of the economy of life with relation to the teeth as organs of mastication.

